# Implementation and Evaluation of a Fully Automated Multiplex Real-Time PCR Assay on the BD Max Platform to Detect and Differentiate Herpesviridae from Cerebrospinal Fluids

**DOI:** 10.1371/journal.pone.0153991

**Published:** 2016-04-19

**Authors:** Thomas Köller, Daniel Kurze, Mirjam Lange, Martin Scherdin, Andreas Podbielski, Philipp Warnke

**Affiliations:** Institute of Medical Microbiology, Virology, and Hygiene, Rostock University Hospital, Rostock, Germany; University of Pittsburgh School of Medicine, UNITED STATES

## Abstract

A fully automated multiplex real-time PCR assay—including a sample process control and a plasmid based positive control—for the detection and differentiation of herpes simplex virus 1 (HSV1), herpes simplex virus 2 (HSV2) and varicella-zoster virus (VZV) from cerebrospinal fluids (CSF) was developed on the BD Max platform. Performance was compared to an established accredited multiplex real time PCR protocol utilizing the easyMAG and the LightCycler 480/II, both very common devices in viral molecular diagnostics. For clinical validation, 123 CSF specimens and 40 reference samples from national interlaboratory comparisons were examined with both methods, resulting in 97.6% and 100% concordance for CSF and reference samples, respectively. Utilizing the BD Max platform revealed sensitivities of 173 (CI 95%, 88–258) copies/ml for HSV1, 171 (CI 95%, 148–194) copies/ml for HSV2 and 84 (CI 95%, 5–163) copies/ml for VZV. Cross reactivity could be excluded by checking 25 common viral, bacterial and fungal human pathogens. Workflow analyses displayed shorter test duration as well as remarkable fewer and easier preparation steps with the potential to reduce error rates occurring when manually assessing patient samples. This protocol allows for a fully automated PCR assay on the BD Max platform for the simultaneously detection of herpesviridae from CSF specimens. Singular or multiple infections due to HSV1, HSV2 and VZV can reliably be differentiated with good sensitivities. Control parameters are included within the assay, thereby rendering its suitability for current quality management requirements.

## Introduction

Herpesviridae like Herpes simplex virus 1 (HSV1), herpes simplex virus 2 (HSV2) and varicella zoster virus (VZV) are causative agents of skin or mucosal tissues lesions as well as of life-threatening infections of the central nervous system (CNS) [1–3]. HSV1 and VZV are among the most important pathogens causing viral encephalitis, HSV2 most notably meningoencephalitis and meningitis in neonates [2,4–8]. For the three species, mortality and morbidity is correlated to appropriate and timely virostatic treatment [3,9–11]. Therefore, a fast detection and discrimination of these 3 pathogens from cerebrospinal fluids (CSF) is crucial for optimum therapy.

PCR based diagnostics allow for a fast and sensitive detection of an increasing range of infectious agents and specimens [10]. Several commercially available and certified test-systems based on diverse protocols are available for separate HSV1, HSV2 and VZV detection from various clinical specimens [12]. In-house multiplex assays covering the simultaneous detection of up to 8 herpesviridae have been published [13–16]. For all these tests, numerous time consuming and error-prone manual working steps are necessary, i.e. preparation of working solutions, DNA extraction, transfer of DNA and mastermixes to the amplification devices.

These technical drawbacks can be circumvented by the usage of fully automated PCR platforms. Recently, protocols for monoplex real-time PCR testing of HSV1, HSV2 and VZV from skin and mucosal lesions as well as duplex testing of HSV1/2 and VZV from various clinical specimens have been published for the BD Max system (Becton Dickinson, Heidelberg, Germany) [17,18].

The present work was aimed to implement and evaluate a multiplex PCR assay on the BD Max platform to simultaneously detect and specifically differentiate HSV1, HSV2, and VZV in CSF specimens while continuously fulfilling quality management requirements.

## Materials and Methods

### Clinical specimens

For clinical validation 123 CSF specimens were analyzed for the presence of HSV1, HSV2, and VZV genomes. Samples were routinely obtained by the attending physician according to clinical indications as well as standard diagnostic algorithms established in this hospital. Samples were collected between 2013–2015 and stored at -80°C. 11 CSF samples previously found to be negative were spiked 1:7 v/v with HSV1 (IC accession: 363055, undiluted = 151500 copies/ml) or HSV2 (IC accession: 363043, undiluted = 28850 copies/ml) positive reference materials from Instand e.V. interlaboratory comparisons (S1 Table).

### Ethics statement

All clinical investigation has been conducted according to the principles expressed in the Declaration of Helsinki. The study was approved by the institutional review board of Rostock University Hospital (Ethikkommission an der Medizinischen Fakultät der Universität Rostock; approval no: A 2015–0161) in line with national and ICH-GCP guidelines. All patients provided written informed consent.

### Reference specimens

For performing quality controls, defining detection limits, checking potential cross reactivity and spiking of negative CSV specimens, samples from the Instand e.V. national interlaboratory comparisons association collected between 2012 and 2015 were employed (http://www.instandev.de). Corresponding accession numbers (IC accession) are displayed for each sample (see section “results”).

### Primers and Probes

Primers and probes were used as previously described [19,20]. Briefly, HSV1 primers and probe were directed to the genomic junction between the glycoprotein G and J genes (nt 137680–137742, accession no. X14112) [20], HSV2 primers and probe to the glycoprotein G gene (nt 139824–139896, accession no. NC_001798) [20] and VZV primers and probe to the glycoprotein B gene (nt 57634–57689, accession no. JN704710). The VZV probe contains incorporated locked nucleic acids (LNAs) to improve the sensitivity and specificity of this short probe [21–23]. All primers and probes were purchased from an accredited commercial provider (Tib Molbiol Syntheselabor GmbH, Germany) (Table 1). *In silico* analysis (Genbank, NCBI) of the primer-probe-sets showed no cross-homology to non-targeted DNA-sequences.

**Table 1:** Primers and probes.

Name	Sequence	T_m_/°C (GC/%)
HSV1	Forward: GGCCTGGCTATCCGGAGA	59.1 (66.7)
	Reverse: GCGCAGAGACATCGCGA	58.8 (64.7)
	Probe: 6FAM-CAGCACACGACTTGGCGTTCTGTGT--BBQ	68.6 (56.6)
HSV2	Forward: GCTCTAGATATCCTCTTTATCATCAGCACC	59.2 (43.3)
	Reverse: TTGTGCTGCCAAGGCGA	60.0 (58.3)
	Probe: YAK-CAGACAAACGAACGCCGCCG--BBQ	68.2 (65.0)
VZV	Forward: TGCAGCGCGGAACTTTTTA	59.1 (47.7)
	Reverse: GCTTCCAGTTCCAACCAACC	58.6 (55.0)
	Probe: ROX-TCACGCCT+CA+T+T+T+AA--BBQ	66.0 (40.0)

Abbreviations: HSV1, herpes simplex virus 1; HSV2, herpes simplex virus 2; VZV, varicella-zoster virus; FAM, 6-carboxyfluorescein; YAK, Yakima Yellow; ROX, X-rodamin; BBQ, black berry quencher; T_m_/°C melting temperature; GC/%, relative guanidine and cytosine content; +C, +T, +A, locked nucleic acids (LNAs).

### Routine diagnostic assay for the detection of HSV1, HSV2, VZV

The routine diagnostic assay is carried out in the DAkkS (Deutsche Akkreditierungsstelle GmbH) accredited laboratory of the Institute of Medical Microbiology, Virology and Hygiene of the University Hospital of Rostock. HSV 1, HSV2 and VZV are detected together with EBV, CMV, HHV6 and a Sample Process Control (SPC) within one test run that is partitioned into two qualitative duplex PCRs (HSV1/HSV2, VZV/HHV6) and one quantitative triplex PCR (EBV/CMV/SPC). The standard extraction step is performed with 200 μl aliquots of patient samples using the NucliSens easyMAG instrument with the Specific B protocol as stated by the manufacturer (BioMérieux, Nürtingen, Germany). The elution volume of extracted sample is 50 μl from which 10 μl are subsequently applied to individual PCR assays. For amplification/detection the LightCycler 480 Probes Master and the LightCycler 480/II are used (Roche, Mannheim, Germany). Amplification mixtures and cycling conditions were adopted from previous publications with minor modifications [19,20,24–27]. This method has an LoD of about 200 copies/ml. It has been validated and accredited and is now used for routine diagnostics of various clinical specimen including CSF.

### BD Max assay

The assay was designed as a fully automated multiplex real-time PCR test including DNA extraction, PCR and results interpretation. DNA was extracted from 400 μl patient samples using the BD MAX^™^ ExK^™^ DNA-2 extraction Kit. The BD Max settings were as follows: The “Master Mix Format” was “Type 2 using BD MMK or BD MMK (SPC) with liquid primers and probes” (BD, Heidelberg, Germany). The BD MMK (SPC) master mix, including all required ingredients as well as a *Drosophila melanogaster* sample process control (SPC), was used with standard extraction parameters. Standard color compensation settings were used.

PCR was performed as follows: initial single step.: 600 s at 98°C, type “hold”; cycle process.: 5s at 98°C and 41.9 s at 60°C type “2-temperature”, 45 cycles. Detection parameters for channel 475/520, 530/565 and 680/715 were adjusted in the following way: “gain”: 40, “Threshold”: 200. For channel 585/630 “gain” was adjusted to 30 and “Threshold”: 200. Signals with intensities below 200 were assessed as “negative”, intensities above 200 were evaluated as “positive”.

The primers/probes mix consisted of 20 pmol of each primer, 10 pmol of each probe, 16% v/v BD Max Diluent (BD, Heidelberg, Germany) and was adjusted to 12.5 μl with nuclease free water (Promega, Madison, WI USA). The mix was subsequently aliquoted in BD Max snap-in tubes, sealed using the Axygen Plate-Max semiautomated plate sealer (Axygen Scientific, Union City, CA, USA) (8s at 180°C), and stored at -20°C until use. The mixture was shown to be stable for at least 3 months but was generally used within 2 weeks.

### Controls

To ensure the adequacy of the test runs, SPC and plasmid based positive controls were used. The SPC, already included within the extraction stripes and the BD MMK (SPC) Mastermix, confirms the quality of the extraction procedure and the suitability of the analyzed patient material. Negative samples lacking the SPC signal were assessed to contain inhibitory factors resulting in non evaluable PCR results. A plasmid based positive control (PC) containing 10^5^ copies/ml of commercially available plasmids carrying amplification targets of each virus was employed to ensure the functional stability of the frozen primer/probes (GenExpress Gesellschaft für Proteindesign mbH, Germany, Table 2). It was included in each test run using an additional reagent strip.

**Table 2:** Plasmids used as positive control.

Name	PCR Fragment length/bp	Plasmid accession no.*
HSV1	217 bp	30-8831-01
HSV2	166 bp	30-8724-01
VZV	240 bp	30-8832-01

Abbreviations: HSV1, herpes simplex virus 1; HSV2, herpes simplex virus 2; VZV, varicella-zoster virus; bp, base pair;

*all plasmids were ordered at GenExpress (GenExpress Gesellschaft für Proteindesign mbH, Germany).

### Measurement of test duration

**Fig 1:**
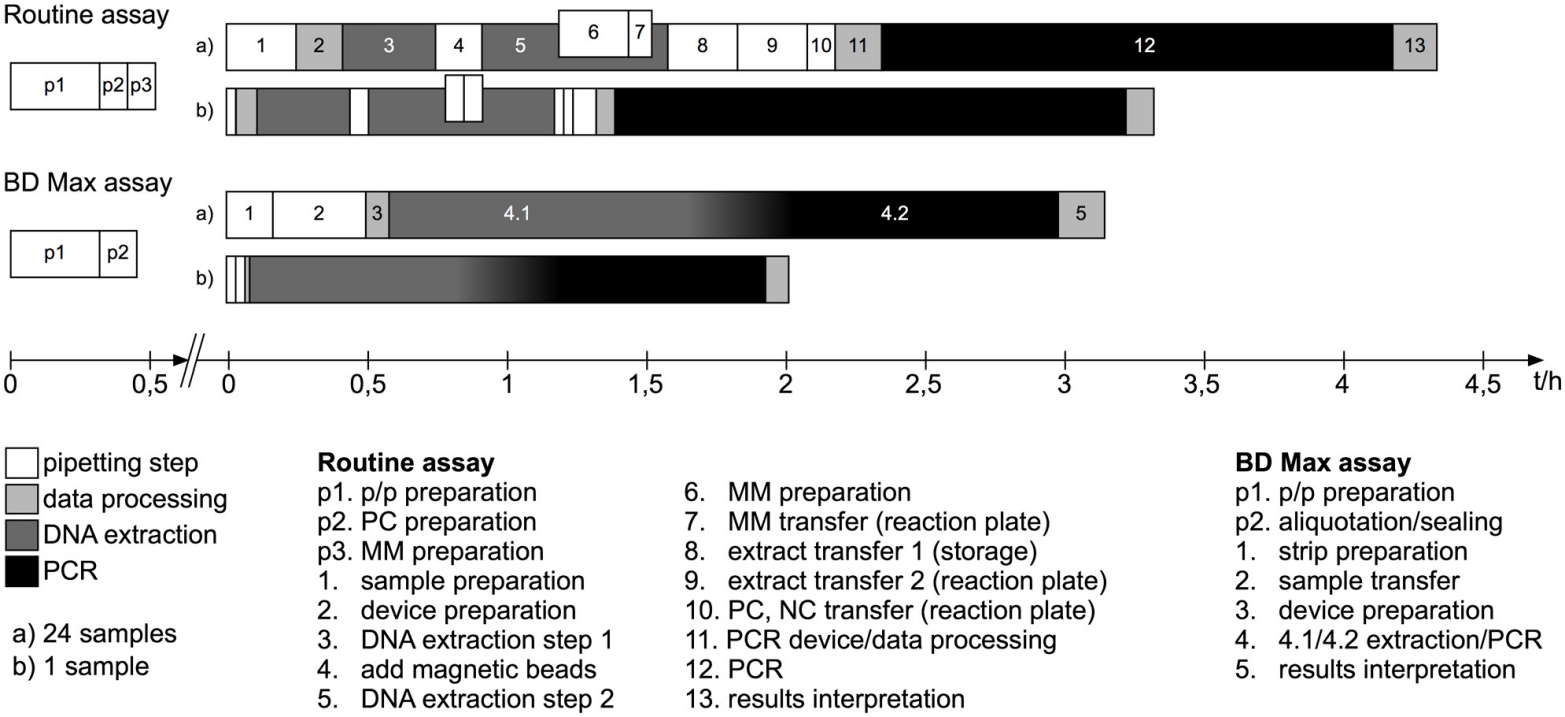
Scheme of workflow. Abbreviations: p/p, primers/probes; PC, positive control; MM, master mix; NC, negative control.

Total time as well as hands-on time was recorded from the beginning of the procedures until the final evaluation of the results (Fig 1). Time was averaged from the work of different technicians (Fig 1). Only test runs containing the same amount of samples were incorporated into the analysis. The preparation of master mixes as well as primer/probes mixes were not included into the work flow analysis since these steps (p1, p2, p3) are inherent part of the analysis protocol.

### Reproducibility

Reproducibility of the results was checked by intra- and interassay analysis. Tests including 3 positive samples of each specimen were performed 3 times within the same run and 3 times in different runs.

### Sensitivity/LoD, Statistical analysis

Serial dilutions of the Instant e.V. reference samples in previously negative tested CSF were used to determine the analytical sensitivity (LoD) of the BD Max assay. Statistical analyses of CI and COV were performed from determined C_T_ values. For means interval estimates in terms of 95%-confidence intervals were specified to provide an information about extent of uncertainty inherent in results derived from data that are themselves only a randomly selected subset of a population. If necessary, lower bound was stated as zero.

### Inter-species cross reactivity

To ensure specificity of the HSV1, HSV2, VSV assay, 25 viral, bacterial and fungal species (S2 Table) typically involved in systemic as well as CNS infections were examined with the above described test format. All species were purchased from ATCC or Instand e.V. Interlaboratory comparison (S2 Table).

## Results

The study focused on methodic benefits on routine diagnostic workflow, reproducibility, sensitivity, as well as the specificity of the individual species-directed PCR assays and compared the new method with an established accredited laboratory protocol employing.

### Turn around and hands-on time reduction

In comparison to the PCR protocol utilizing the Nuclisens easyMAG and the Roche LightCycler, the new BD Max analysis protocol was found to be about 27%–39% faster, depending on sample numbers (Fig 1). The analysis of 24 samples employing the BD Max system takes about 190 min in average compared to 260 min when utilizing the conventional procedure, for analysis of single samples, 122 min and 200 min, respectively (Fig 1). Again depending on sample numbers, hands-on time on the BD Max was found to be reduced for about 60% to 74% (24 samples: 45 min vs. 111 min, 1 sample 10 min vs. 38 min) as compared to the conventional procedure. Work steps could be reduced from 13 to 5 steps using the BD Max protocol (Fig 1).

### Reproducibility

The c_T_ values of all specimens confirmed reproducibility within a coefficient of variation between 0.4%–2.1% in intra-assay and between 0.6%–3.2% in inter-assay analysis (Table 3).

**Table 3:** Reproducibility.

Virus	Sample	Intraassay MV ± SD (COV/%)	Interassay MV ± SD (COV/%)
HSV1	1	23.5 ± 0.5 (2.1)	24.0 ± 0.5 (1.9)
	2	16.2 ± 0.2 (1.2)	16.3 ± 0.4 (2.5)
	3	19.8 ± 0.4 (1.8)	20.1 ± 0.3 (1.5)
HSV2	11	29.9 ± 0.1 (0.4)	30.0 ± 0.7 (2.2)
	12	34.0 ± 0.3 (0.9)	33.8 ± 0.2 (0.6)
	13	33.3 ± 1.0 (3.0)	32.6 ± 1.1 (3.2)
VZV	29	27.1 ± 0.5 (1.7)	27.2 ± 0.2 (0.6)
	30	28.7 ± 0.5 (1.8)	29.5 ± 0.9 (3.2)
	35	29.2 ± 0.6 (1.9)	28.1 ± 0.6 (2.0)

Abbreviations: MV, mean c_t_ value; SD, standard deviation; COV, coefficient of variation; for sample numbers please refer to S1 Table.

### Sensitivity/LoD, Statistical analysis

The LoD of the BD Max multiplex assay was found to be 173 copies/ml (95% CI, 88–258) for HSV1, 171 copies/ml (95% CI, 148–194) for HSV 2, and 84 copies/ml (95% CI, 5–163) for VZV, respectively (Table 4).

**Table 4:** Limit of detection (LoD).

Virus (IC no.)	copies/ml (95% CI)	MV ± SD (COV/%)	Detection rate
HSV1 (363040)	346 (292–400)	33.68 ± 0.36 (1.06)	5:5 (100%)
	173 (88–258)	34.36 ± 0.56 (1.64)	5:5 (100%)
	86 (0–193)	35.18 ± 0.63 (1.80)	4:5 (80%)
HSV2 (363047)	342 (325–359)	35.84 ± 0.59 (1.67)	5:5 (100%)
	171 (148–194)	40.48 ± 0.83 (2.06)	5:5 (100%)
	85 (58–112)	43.90 ± 0.75 (1.72)	3:5 (60%)
VZV (366031)	338 (309–367)	35.14 ± 0.55 (1.58)	5:5 (100%)
	169 (144–194)	36.00 ± 0.47 (1.32)	5:5 (100%)
	84 (5–163)	38.80 ± 1.50 (3.88)	5:5 (100%)

Abbreviations: IC no., Interlaboratory comparison number; MV, mean c_t_ value; SD, standard deviation; COV, coefficient of variation; 95% CI, confidence interval.

### Specificity

No cross reaction was observed between HSV1, HSV2, and VZV positive samples investigating patient—and reference materials (S1 and S2 Tables). Inter-species cross reactivity could be excluded for 25 common viral, bacterial and fungal species (S2 Table).

### Clinical validation

123 clinical CSF specimens were tested utilizing both, the BD Max system and the established method using the easyMAG in combination with the Lightcycler 480/II. In all, the observed concordance between the two methods was 97.6% for CSF specimens. 3 samples showed negative SPC detection resulting in non-evaluable PCR data. (Table 5, S2 Table).

**Table 5:** Summary of CSF analyses with BD Max.

Specimens	Positive (%)			Negative (%)	Inhibited (%)
	HSV1	HSV2	VZV	all	all
CSF	5 (4.1)	4 (3.3)	18 (14.6)	82 (66.7)	3 (2.4)
spiked CSF	5 (4.1)	6 (4.9)	-	-	-
all	10 (8.2)	10 (8.2)	18 (14.6)	82 (66.7)	3 (2.4)

Abbreviations: CSF, cerebrospinal fluid; spiked CSF, HSV1 or HSV2 enriched cerebrospinal fluid.

### Interlaboratory comparison

40 positive Instand e.V. reference samples were used to check the validity of the new protocol in comparison with both, our in-house method and the officially released results of the Instand e. V. national interlaboratory tests. All results were 100% concordant with the reference results (Table 6).

**Table 6:** Interlaboratory comparison/reference samples.

No.	IC-accession	expected result (copies/ml)	BD Max result (c_t_)	Routine PCR result (C_p_)
1	363030	HSV1 (45625)	HSV1 (28.9)	HSV1 (27.2)
2	363035	HSV1 (1236509)	HSV1 (27.3)	HSV1 (25.9)
3	363037	HSV1 (98250)	HSV1 (28.2)	HSV1 (23.15)
4	363040	HSV1 (52000)	HSV1 (29.2)	HSV1 (26.4)
5	363042	HSV1 (14500)	HSV1 (29.8)	HSV1 (27.6)
6	363048	HSV1 (18224)	HSV1 (29.8)	HSV1 (28.4)
7	363051	HSV1 (17100)	HSV1 (30.2)	HSV1 (31)
8	363053	HSV1 (45917)	HSV1 (28.1)	HSV1 (29.6)
9	363055	HSV1 (151500)	HSV1 (26.9)	HSV1 (24.3)
10	363057	HSV1 (146175)	HSV1 (27.3)	HSV1 (26.1)
11	363025	HSV2 (33600)	HSV2 (29.3)	HSV2 (29.9)
12	363029	HSV2 (10975)	HSV2 (30.9)	HSV2 (31.8)
13	363033	HSV2 (14000)	HSV2 (30.2)	HSV2 (31.6)
14	363036	HSV2 (4500)	HSV2 (31.8)	HSV2 (30.9)
15	363038	HSV2 (45180)	HSV2 (28.7)	HSV2 (27.54)
16	363039	HSV2 (14225)	HSV2 (30.7)	HSV2 (29.6)
17	363043	HSV2 (28850)	HSV2 (29.5)	HSV2 (28.6)
18	363045	HSV2 (7784)	HSV2 (31.2)	HSV2 (30.7)
19	363047	HSV2 (51500)	HSV2 (28.9)	HSV2 (27.6)
20	363050	HSV2 (11255)	HSV2 (30.1)	HSV2 (30.9)
21	366017	VZV (88000)	VZV (29.3)	VZV (28.1)
22	366022	VZV (71200)	VZV (29.8)	VZV (28.78)
23	366024	VZV (22900)	VZV (30.5)	VZV (30.6)
24	366026	VZV (56700)	VZV (30.2)	VZV (27.55)
25	366030	VZV (24250)	VZV (30.2)	VZV (27.8)
26	366031	VZV (50825)	VZV (29.6)	VZV (28.6)
27	366033	VZV (43500)	VZV (29.5)	VZV (28.2)
28	366036	VZV (21510)	VZV (30.4)	VZV (29)
29	366038	VZV (29200)	VZV (30.3)	VZV (28.7)
30	366042	VZV (40000)	VZV (29.6)	VZV (28.3)
31	363034	negative (0)	negative (-)	negative (-)
32	363026	negative (0)	negative (-)	negative (-)
33	363044	negative (0)	negative (-)	negative (-)
34	363054	negative (0)	negative (-)	negative (-)
35	363041	negative (0)	negative (-)	negative (-)
36	366023	negative (0)	negative (-)	negative (-)
37	366018	negative (0)	negative (-)	negative (-)
38	366029	negative (0)	negative (-)	negative (-)
39	366034	negative (0)	negative (-)	negative (-)
40	366043	negative (0)	negative (-)	negative (-)

Abbreviations: IC accession, accession numbers of Instand e.V. interlaboratory comparison.

## Discussion

HSV1, HSV2 and VZV are common pathogens causing severe CNS infections [1–3]. Numerous in-house protocols and commercially available “CE”-certified platforms such as the “Lyra^®^ Direct HSV1+2/VZV assay” (Quidel Germany GmbH, Germany), the “RealStar^®^ alpha Herpesvirus PCR” Kit (Altona Diagnostics, Germany) and the “HSV-1 HSV-2 VZV R-gene^®^ kit” (Argene/bioMerieux, France) are available for their detection, but all of these tests are either not approved for CSF or cannot be run with full automation [12,28–32].

The introduced, fully automated multiplex real time PCR assay on the BD Max platform allows for the simultaneous detection and specific differentiation of HSV1, HSV2 and VZV from cerebrospinal fluids. Quality parameters, demanded for example by quality management systems, are covered by including sample process and positive controls. Manual preparation steps, hands-on time and total processing time could be significantly decreased as compared to a conventional Nuclisens easyMAG and Roche LightCycler based protocol, facilitating usage in routine diagnostics. The simplified workflow enables a more variable routine work plan especially in cases of additionally requested emergency diagnostics. This in combination with the complete automation could reduce flaws in sample preparation inevitable present when manual handling is performed.

Prior studies pointed out low HSV1-, HSV2- and VZV-DNA concentrations in CSF in cases of acute encephalitis and meningitis [12,33–42]. Therefore, HSV1/2 and VZV detection levels of approximately 200 copies/ml are considered to be necessary for adequate diagnostics [43]. The LoD of the present PCR protocol clearly fulfills these postulated sensitivity criteria.

The comparison of 123 CSF specimens prospectively analyzed with the established routine PCR and retrospectively investigated with the new protocol revealed a 97.6% concordance. In comparison to other methodical studies the discordance of 2.4% is within the range normally found when comparing different herpesviridae PCR protocols [17,40,44–46].

Cardenas and colleagues established a HSV1, HSV2 and VZV assay on the BD Max platform for specimens from skin and mucosal lesions as a proof of principle to demonstrate the BD Max to be a suitable one-step open-access-platform [18]. In contrast to the present work they established two different protocols to detect HSV1 and HSV2 as duplex—and VZV separately as a singleplex PCR. This approach does not cover the technical potential of the BD Max to analyze up to 5 PCR targets within one test, and thus, increases the material costs and complicates the workflow. Furthermore, the required amounts of patient material plays a minor role when using skin or mucosal swabs, but could be a major obstacle for CSF diagnostics.

Pillet and colleagues published an excellent protocol to detect HSV and VZV from various specimens including 38 CSFs utilizing the BD Max platform [17]. In comparison to their protocol the present method contains some features leading to improved analytical value and feasibility, like specific differentiation between HSV1 and 2, usage of ready-made mastermixes and positive controls. Despite similar clinical manifestations, the discrimination between HSV1 and HSV2 is important since CNS infection site, potential complications, responses and resistance rates to antivirals, as well as reactivation rates on mucosal surfaces vary between both virus types. Therefore, a HSV-type-specific test result leads to a more detailed statement on the infection status and its prognosis [47,48]. The described utilization of the commercially available pre-assembled BD MMK(SPC) mastermix leads to a decreased labor input for sample preparation and simplifies the quality management processes since the tubes are delivered with lyophilized aliquots and with information on batches as well as expiry dates. The additional use of plasmid based PC improves validity of negative results ensuring the functionality of the in-house prealiquoted primer/probes mixes.

Recently, FilmArray Meningitis/Encephalitis Panel, a cartridge based fully automated multiplex PCR-system was introduced allowing for the detection of 14 pathogens, including HSV1, HSV2 and VZV, requiring little hands-on and turn-around time (BioFire, USA). In comparison to the presented BD Max assay, the LODs achieved with the FilmArray system for these viruses range from 1290 to 1660 copies/ml. This is approximately 7.5–19.7 times higher than the LODs achieved by the presented method. This potentially could lead to false negative results, especially when dealing with low viral loads.

In conclusion, this new method is the first fully automated multiplex assay for the simultaneous detection and differentiation of HSV1, HSV2, and VZV from CSF specimens on the BD Max platform. The combined usage of a positive and a sample process control covers relevant quality interests. Complete method validation including 123 clinical CSF specimens gives a profound and reliable overview about the test performance in routine settings.

## Supporting Information

S1 Table CSF from patients. Abbreviations: CSF, cerebrospinal fluid; spiked CSF, HSV1 or HSV2 enriched CSF.
(DOCX)

S2 Table Specificity / cross reactivity. *: accession numbers of Instand e.V. interlaboratory comparison, ATCC or DSM.
(DOCX)
