# Supplementary material for: Implementation and Evaluation of a Fully Automated Multiplex Real-Time PCR Assay on the BD Max Platform to Detect and Differentiate Herpesviridae from Cerebrospinal Fluids
Source: PLoS One. 2016 Apr 19;11(4):e0153991. doi: 10.1371/journal.pone.0153991 (PMC4836685; doi:10.1371/journal.pone.0153991)
Supplement: S1 Table — Abbreviations: CSF, cerebrospinal fluid; spiked CSF, HSV1 or HSV2 enriched CSF. (DOCX) [file pone.0153991.s001.docx]

**S1 Table. CSF from patients.**

| **No.** | **Specimen** | **HSV1** | **HSV2** | **VZV** | **SPC** | **Result** | **matching routine PCR** |
| --- | --- | --- | --- | --- | --- | --- | --- |
| 1 | CSF | 23.1 | - | - | 27.0 | HSV1 | yes |
| 2 | CSF | 27.3 | - | - | 27.5 | HSV1 | yes |
| 3 | CSF | 29.9 | - | - | 28.0 | HSV1 | yes |
| 4 | CSF | 31.6 | - | - | 28.3 | HSV1 | yes |
| 5 | CSF | 31.8 | - | - | 26.7 | HSV1 | yes |
| 6 | Spiked CSF | 28.7 | - | - | 28.0 | HSV1 | yes |
| 7 | Spiked CSF | 29.1 | - | - | 28.2 | HSV1 | yes |
| 8 | Spiked CSF | 29.5 | - | - | 29.0 | HSV1 | yes |
| 9 | Spiked CSF | 29.7 | - | - | 28.8 | HSV1 | yes |
| 10 | Spiked CSF | 30.0 | - | - | 29.8 | HSV1 | yes |
| 11 | CSF | - | 25.4 | - | 27.6 | HSV2 | yes |
| 12 | CSF | - | 28.9 | - | 28.0 | HSV2 | yes |
| 13 | CSF | - | 29.3 | - | 27.6 | HSV2 | yes |
| 14 | CSF | - | 41.3 | - | 27.6 | HSV2 | yes |
| 15 | Spiked CSF | - | 32.5 | - | 28.5 | HSV2 | yes |
| 16 | Spiked CSF | - | 32.6 | - | 30.6 | HSV2 | yes |
| 17 | Spiked CSF | - | 32.8 | - | 28.3 | HSV2 | yes |
| 18 | Spiked CSF | - | 32.8 | - | 28.4 | HSV2 | yes |
| 19 | Spiked CSF | - | 32.9 | - | 29.8 | HSV2 | yes |
| 20 | Spiked CSF | - | 33.4 | - | 34.1 | HSV2 | yes |
| 21 | CSF | - | - | 42.8 | 27.0 | VZV | yes |
| 22 | CSF | - | - | 31.9 | 27.2 | VZV | yes |
| 23 | CSF | - | - | 33.3 | 26.8 | VZV | yes |
| 24 | CSF | - | - | 31.0 | 27.8 | VZV | yes |
| 25 | CSF | - | - | 30.2 | 26.9 | VZV | yes |
| 26 | CSF | - | - | 32.5 | 27.0 | VZV | yes |
| 27 | CSF | - | - | 32.3 | 27.2 | VZV | yes |
| 28 | CSF | - | - | 35.7 | 27.5 | VZV | yes |
| 29 | CSF | - | - | 24.7 | 30.2 | VZV | yes |
| 30 | CSF | - | - | 30.3 | 32.8 | VZV | yes |
| 31 | CSF | - | - | 38.3 | 33.8 | VZV | yes |
| 32 | CSF | - | - | 42.8 | 31.0 | VZV | yes |
| 33 | CSF | - | - | 34.0 | 28.5 | VZV | yes |
| 34 | CSF | - | - | 42.2 | 29.1 | VZV | yes |
| 35 | CSF | - | - | 30.9 | 28.6 | VZV | yes |
| 36 | CSF | - | - | 36.1 | 28.9 | VZV | yes |
| 37 | CSF | - | - | 34.7 | 29.0 | VZV | yes |
| 38 | CSF | - | - | 30.6 | 28.6 | VZV | yes |
| 39 | CSF | - | - | - | 27.9 | negative | yes |
| 40 | CSF | - | - | - | 28.8 | negative | yes |
| 41 | CSF | - | - | - | 29.6 | negative | yes |
| 42 | CSF | - | - | - | 32.3 | negative | yes |
| 43 | CSF | - | - | - | 29.9 | negative | yes |
| 44 | CSF | - | - | - | 28.4 | negative | yes |
| 45 | CSF | - | - | - | 28.6 | negative | yes |
| 46 | CSF | - | - | - | 28.4 | negative | yes |
| 47 | CSF | - | - | - | 28.7 | negative | yes |
| 48 | CSF | - | - | - | 31.1 | negative | yes |
| 49 | CSF | - | - | - | 30.0 | negative | yes |
| 50 | CSF | - | - | - | 30.7 | negative | yes |
| 51 | CSF | - | - | - | 29.3 | negative | yes |
| 52 | CSF | - | - | - | 29.0 | negative | yes |
| 53 | CSF | - | - | - | 24.0 | negative | yes |
| 54 | CSF | - | - | - | 29.3 | negative | yes |
| 55 | CSF | - | - | - | 28.7 | negative | yes |
| 56 | CSF | - | - | - | 28.9 | negative | yes |
| 57 | CSF | - | - | - | 28.3 | negative | yes |
| 58 | CSF | - | - | - | 31.4 | negative | yes |
| 59 | CSF | - | - | - | 29.3 | negative | yes |
| 60 | CSF | - | - | - | 29.7 | negative | yes |
| 61 | CSF | - | - | - | 28.9 | negative | yes |
| 62 | CSF | - | - | - | 29.3 | negative | yes |
| 63 | CSF | - | - | - | 28.8 | negative | yes |
| 64 | CSF | - | - | - | 27.7 | negative | yes |
| 65 | CSF | - | - | - | 29.6 | negative | yes |
| 66 | CSF | - | - | - | 27.9 | negative | yes |
| 67 | CSF | - | - | - | 27.8 | negative | yes |
| 68 | CSF | - | - | - | 29.8 | negative | yes |
| 69 | CSF | - | - | - | 28.3 | negative | yes |
| 70 | CSF | - | - | - | 28.5 | negative | yes |
| 71 | CSF | - | - | - | 28.0 | negative | yes |
| 72 | CSF | - | - | - | 28.2 | negative | yes |
| 73 | CSF | - | - | - | 27.9 | negative | yes |
| 74 | CSF | - | - | - | 28.0 | negative | yes |
| 75 | CSF | - | - | - | 30.0 | negative | yes |
| 76 | CSF | - | - | - | 27.6 | negative | yes |
| 77 | CSF | - | - | - | 30.4 | negative | yes |
| 78 | CSF | - | - | - | 28.6 | negative | yes |
| 79 | CSF | - | - | - | 30.1 | negative | yes |
| 80 | CSF | - | - | - | 29.0 | negative | yes |
| 81 | CSF | - | - | - | 29.3 | negative | yes |
| 82 | CSF | - | - | - | 29.7 | negative | yes |
| 83 | CSF | - | - | - | 28.9 | negative | yes |
| 84 | CSF | - | - | - | 28.2 | negative | yes |
| 85 | CSF | - | - | - | 29.1 | negative | yes |
| 86 | CSF | - | - | - | 29.5 | negative | yes |
| 87 | CSF | - | - | - | 29.2 | negative | yes |
| 88 | CSF | - | - | - | 29.5 | negative | yes |
| 89 | CSF | - | - | - | 28.7 | negative | yes |
| 90 | CSF | - | - | - | 30.1 | negative | yes |
| 91 | CSF | - | - | - | 36.4 | negative | yes |
| 92 | CSF | - | - | - | 29.8 | negative | yes |
| 93 | CSF | - | - | - | 30.0 | negative | yes |
| 94 | CSF | - | - | - | 32.5 | negative | yes |
| 95 | CSF | - | - | - | 34.1 | negative | yes |
| 96 | CSF | - | - | - | 30.1 | negative | yes |
| 97 | CSF | - | - | - | 29.9 | negative | yes |
| 98 | CSF | - | - | - | 27.4 | negative | yes |
| 99 | CSF | - | - | - | 30.1 | negative | yes |
| 100 | CSF | - | - | - | 35.7 | negative | yes |
| 101 | CSF | - | - | - | 29.7 | negative | yes |
| 102 | CSF | - | - | - | 29.9 | negative | yes |
| 103 | CSF | - | - | - | 30.9 | negative | yes |
| 104 | CSF | - | - | - | 29.1 | negative | yes |
| 105 | CSF | - | - | - | 29.7 | negative | yes |
| 106 | CSF | - | - | - | 29.3 | negative | yes |
| 107 | CSF | - | - | - | 29.0 | negative | yes |
| 108 | CSF | - | - | - | 29.2 | negative | yes |
| 109 | CSF | - | - | - | 28.8 | negative | yes |
| 110 | CSF | - | - | - | 27.9 | negative | yes |
| 111 | CSF | - | - | - | 28.3 | negative | yes |
| 112 | CSF | - | - | - | 29.0 | negative | yes |
| 113 | CSF | - | - | - | 28.2 | negative | yes |
| 114 | CSF | - | - | - | 27.8 | negative | yes |
| 115 | CSF | - | - | - | 30.5 | negative | yes |
| 116 | CSF | - | - | - | 29.3 | negative | yes |
| 117 | CSF | - | - | - | 28.0 | negative | yes |
| 118 | CSF | - | - | - | 29.8 | negative | yes |
| 119 | CSF | - | - | - | 28.4 | negative | yes |
| 120 | CSF | - | - | - | 29.1 | negative | yes |
| 121 | CSF | - | - | - | inhibition | inhibition | no |
| 122 | CSF | - | - | - | inhibition | inhibition | no |
| 123 | CSF | - | - | - | inhibition | inhibition | no |

Abbreviations: CSF, cerebrospinal fluid; spiked CSF, HSV1 or HSV2 enriched CSF
