# Supplementary material for: Implementation and Evaluation of a Fully Automated Multiplex Real-Time PCR Assay on the BD Max Platform to Detect and Differentiate Herpesviridae from Cerebrospinal Fluids
Source: PLoS One. 2016 Apr 19;11(4):e0153991. doi: 10.1371/journal.pone.0153991 (PMC4836685; doi:10.1371/journal.pone.0153991)
Supplement: S2 Table — *: accession numbers of Instand e.V. interlaboratory comparison, ATCC or DSM. (DOCX) [file pone.0153991.s002.docx]

**S2 Table. Specificity / cross reactivity.**

| **sample no.** | **accession no.*** | **species** | **result HSV1/HSV2/VZV** |
| --- | --- | --- | --- |
| 1 | 371036 | Adenovirus | negative |
| 2 | 365071 | Cytomegalovirus | negative |
| 3 | 372040 | Echovirus 7 | negative |
| 4 | 372041 | Enterovirus 68 | negative |
| 5 | 376036 | Epstein-Barr virus | negative |
| 6 | 394001 | Human Polyomavirus 2 | negative |
| 7 | 300049 | Influenza A virus | negative |
| 8 | 300050 | Influenza B virus | negative |
| 9 | 367072 | Parvovirus B19 | negative |
| 10 | ATCC 13048 | *E. aerogenes* | negative |
| 11 | ATCC 25922 | *E. coli* | negative |
| 12 | ATCC BAA 1152 | *E. corrodens* | negative |
| 13 | ATCC 51299 | *E. faecalis* | negative |
| 14 | ATCC 49247 | *H. influenzae* | negative |
| 15 | ATCC 700324 | *K. oxytoca* | negative |
| 16 | ATCC 700603 | *K. pneumoniae* | negative |
| 17 | ATCC 13077 | *N. meningitidis* | negative |
| 18 | ATCC 27853 | *P. aeruginosa* | negative |
| 19 | ATCC 13813 | *S. agalactiae* | negative |
| 20 | ATCC 29213 | *S. aureus* | negative |
| 21 | DSM 1798 | *S. epidermidis* | negative |
| 22 | ATCC 49619 | *S. pneumoniae* | negative |
| 23 | ATCC 12344 | *S. pyogenes* | negative |
| 24 | ATCC 14053 | *C. albicans* | negative |
| 25 | ATCC NYA 2950 | *C. glabrata* | negative |

*****: accession numbers of Instand e.V. interlaboratory comparison, ATCC or DSM
